# Endovascular and Surgical Options for Ruptured Middle Cerebral Artery Aneurysms: Review of the Literature

**DOI:** 10.1155/2014/315906

**Published:** 2014-07-06

**Authors:** David R. Santiago-Dieppa, Jeffrey S. Pannell, Alexander A. Khalessi

**Affiliations:** Division of Neurosurgery, University of California, San Diego, 200 West Arbor Drive, San Diego, CA 92103, USA

## Abstract

Middle cerebral artery (MCA) aneurysms are common entities, and those of the bifurcation are the most frequently encountered sublocation of MCA aneurysm. MCA bifurcation (MBIF) aneurysms commonly present with subarachnoid hemorrhage (SAH), are devastating, and are often lethal. At the present time, the treatment of ruptured MBIF aneurysms entails either endovascular or open microneurosurgical methods to permanently secure the aneurysm(s). The purpose of this report is to review the current available data regarding the relative superiority of endovascular versus open microneurosurgical clipping for the treatment of ruptured middle cerebral artery bifurcation aneurysms.

## 1. Introduction

Intracranial aneurysm rupture with resultant subarachnoid hemorrhage (aSAH) is a serious and often deadly phenomenon with an incidence that affects as many as 30,000 individuals each year in the United States [1, 2]. When cerebral aneurysms are considered by location, 40% involve the middle cerebral artery (MCA), and when all MCA aneurysms are considered by subtype, those of the MCA bifurcation (MBIF) represent 81% of all cases and 87% of all ruptured MCA aneurysms according to the Kuopio Cerebral Aneurysm Database, one of the largest population based series ever collected [3–6]. Currently, the treatment of ruptured MBIF aneurysms is immensely controversial. At present day, both endovascular coiling and microneurosurgical clipping techniques represent viable treatment modalities [7]. However, the strengths and limitations of the two techniques suggest a complementary relationship; factors including aneurysm morphology and presence of mass effect related to hemorrhage may drive treatment selection.

The history of open surgical clipping dates back to the early 1937, when American neurosurgeon Dandy clipped and secured an aneurysm of the internal carotid artery [8]; it was a pioneering event that many would consider the origin of modern cerebrovascular neurosurgery [9]. Over the ensuing half century following Dandy's report, the neurosurgical management of cerebrovascular disease continued to robustly evolve in both technique and application. Within a matter of just a few decades microneurosurgical clipping became the definite management for permanently securing cerebral aneurysms [6].

In 1991, however, the landscape of cerebrovascular neurosurgery began to change when Guglielmi et al. reported their experience with the first detachable coils [10, 11]. Moreover, as the initial results of the International Subarachnoid Hemorrhage Trial (ISAT) emerged in 2002 demonstrating improved clinical outcomes for endovascular patients [12], the paradigm of cerebral aneurysm treatment began to dramatically shift away from open microneurosurgical techniques.

Today, the endovascular management of cerebral aneurysms is continuing to become more prevalent, as evidenced by the report by van Dijk et al. [13] which examined trends in the management of ruptured cerebral aneurysms from 2002 to 2008 and found a statistically significant increase in the number of endovascular interventions rising from 17.28% to 57.59% of cases. However, despite the clear momentum of endovascular treatment, many question whether it is really superior to microsurgical management [13]. Ultimately, it is not yet fully known which treatment modality is definitively superior, in part because ruptured cerebral aneurysm represents a complex and multifactorial clinical situation that often needs to be considered in the context of sublocation, the size and morphology of the aneurysm, the patients age, and comorbid pathology such as a hydrocephalus and mass-exerting parenchymal hematomas [14].

## 2. Randomized Controlled Trials

To date, there have been no randomized controlled trials (RCTs) that have specifically addressed the issue of surgical clipping versus coil embolization for ruptured MBIF aneurysms. However, there have been 3 randomized controlled trials (RCTs) that have compared open microsurgical clipping to endovascular coiling for the treatment of all locations of ruptured cerebral aneurysms [12, 15–19].

The first RCT originated in Finland and randomized 109 patients with aSAH to either surgery or coil embolization [15]. At 1-year followup, Koivisto et al. reported a good recovery on the Glasgow Outcome Scale (GOS) of 76.9% versus 66.7% for the endovascular and surgical cohorts, respectively [15]. Although the study showed a trend towards improved outcome in the endovascular cohort, the results were not of statistical significance, and no long-term followup was ever reported.

The International Subarachnoid Hemorrhage Trial (ISAT) was the second RCT to address the relative merits of coiling versus clipping and randomized 2143 patients to endovascular versus open microneurosurgical treatment [12]. At 1 year, the results of the ISAT trail showed that clinical outcomes based on the modified Rankin score (mRS) were better in the endovascular treatment group relative to those who had undergone surgical treatment. Furthermore, the ISAT found that a statistically significant 23.7% of endovascular patients were dependent or dead at 1 year as compared to 30.6% of patients randomized to open surgical treatment. Subsequently, the 5-year results of the ISAT showed that the proportion of survivors who were independent with a mRS 0–2 was no longer of significance: 83% versus 82% for the endovascular and neurosurgical groups, respectively [19]. However, the risk of death in patients randomized to coiling continued to remain lower than the risk of death for patients in the surgical cohort (RR 0.77, *P* = 0.03) [19].

The final RCT to address the question of coiling versus clipping was the Barrow Ruptured Aneurysm Trial (BRAT) [17]. This trial was unique in design when compared to the ISAT and involved an intention-to-treat design that included all patients with aSAH [17]. The 1-year results of the BRAT trial showed that the endovascular cohort exhibited a statistically significant lower rate of poor clinical outcome (mRS > 2) as compared to those in the surgical cohort 23.2% versus 33.7%, respectively. Interestingly, the 3-year results of the BRAT found that the 3-year risk of a poor clinical outcome in the surgical clipping versus endovascular group was 35.8% versus 30%, respectively, which was no longer of statistical significance [18]. This finding represented a decrease in the absolute difference between the 2 groups from 10.5% at 1 year to 5.8% at 3 years [18]. Although the BRAT did not include a subgroup analysis of just MBIF aneurysm when all anterior circulation aneurysms alone were analyzed, the risk of a poor clinical outcome in the endovascular versus microneurosurgical clipping group was 25.3% versus 28.5% at 1 year and 29.3% versus 27.9% at 3 years, respectively, indicating that no difference existed between the 2 cohorts [18].

## 3. Retrospective Case Series

There are currently no published case series or reports that specifically address ruptured MBIF aneurysms and the relative benefits of coil embolization versus surgical clipping. However, there are several case series that examine the experiences of endovascular treatment for MCA aneurysms [20–31]. Brinjikji et al. performed a systematic review of the literature involving coiling of all MCA aneurysms and found the combined morbidity and mortality for all subtypes of ruptured MCA aneurysms to be 6% with a complete postoperative occlusion reached in 82.4% of cases which suggested that endovascular treatment was a viable treatment option that rivaled surgical clipping.

More recently, Abla et al. performed a retrospective review of all MCA aneurysms treated at single-intuition over a 5-year period [32]. Of the 149 MCA aneurysms included, surgical clipping was found to be the preferred method of treatment in 115 (77.2%) of cases. Of the remaining MCA aneurysms 22.8% were treated via endovascular techniques, 76.5% of which were MBIF aneurysms [32].

Diaz et al. compared a single institutions experience with 90 consecutive MCA aneurysms treated via open or endovascular methods. They found no statistical difference on angiographic occlusion or clinical outcomes but did find a statistically significant difference when considering retreatment rates and procedural complication rates, both of which were higher in the endovascular group [33].

The case for the microneurosurgical management of MCA aneurysms is strong, and tremendous advances have been made in the field over the past decade [34]. Rodríguez-Hernández et al. recently published a retrospective series from UCSF that reported their experience with 631 MCA aneurysms managed with a “clip first policy.” Of the 631 aneurysms two hundred eighty-two patients presented with ruptured aneurysms. Good outcomes, which were defined as mRS scores of 0–2, were achieved in 198 (70%) of patients. Impressively, 99% of the aneurysms clipped were found to be completely obliterated, which stand in stark contrast to retrospective studies managed by endovascular methods [35].

## 4. Discussion

At the present time the treatment of ruptured MCA aneurysms remains controversial. When all current evidence regarding the treatment of ruptured MBIF aneurysms is considered, it is difficult to generalize the results of the Finnish [15], ISAT [12, 16, 19], and BRAT [17, 18] trials given that no published subgroup analysis addressed specific aneurysmal location. For example, no RCT comparing microneurosurgical clipping to endovascular coiling in wide neck MBIF aneurysms has been conducted. However, despite the paucity of research that specifically addresses the MBIF aneurysms, general principles do exist regarding the relative benefits of surgical and endovascular treatment. In addition to location, it is well known that aneurysm size, patient age, and the presence of medical comorbidities are important risk factors that influence the natural history and affect the patients' ultimate outcome [2, 14, 36].

In 2012, the Stroke Council of the American Heart Association published an update to the 2009 Guidelines for the Management of Aneurysmal Subarachnoid Hemorrhage, [2] in which they issued the following class 1 recommendation [37]:

*“Determination of aneurysm treatment, as judged by both experienced cerebrovascular Surgeons and endovascular specialists, should be a multidisciplinary decision based on characteristics of the patient and the aneurysm. For patients with ruptured aneurysms judged to be technically amenable to both endovascular coiling and microneurosurgical clipping, endovascular coiling should be considered.”*

However, despite the increasing momentum for endovascular treatment, as reflected by the most recent guidelines [2, 37, 38], the general applicability of the ISAT trial results has been called into question [39]. Many authors feel that surgical clipping offers select patients with ruptured MCA aneurysms the best long-term clinical outcome [32, 40–43].

The presence of a large (>50 mL) intraparenchymal hematoma exerting mass effect stands as the clearest indication for surgical evacuation and subsequent microsurgical clipping. More specifically, evacuation within 3.5 hours has been shown to improve outcomes [44]. The Kuopio Cerebral Aneurysm Database that found 44% of all ruptured MCA aneurysms tend to present with ICH [3–6]. In the BRAT trial [17, 18], the frequency of patients crossover from endovascular to surgical treatment was 38% for the total group, 42% for all anterior circulation aneurysms, and 66.77% for the MCA subgroup of anterior circulation aneurysms which illustrates the important role that open surgery continues to play a role in the comprehensive management of MCA aneurysms [17, 18].

The patient's age, the presence of medical comorbidities, and the presenting World Federation of Neurological Surgeons classification (WFNS) grade are important risk factors to consider when deciding on open versus closed surgery. The 2012 Stroke Council of the American Heart Association Guidelines for the Management of Aneurysmal Subarachnoid Hemorrhage issued a class IIb recommendation that patients >70 and those with a poor WFNS grade (IV/V) may be better endovascular candidates [37].

Preservation of the parent artery is crucial when securing a ruptured aneurysm and is of greater significance for MBIF aneurysms given the lack of robust collateral circulation [6]. One clear advantage to open microsurgical management is the ability to directly visualize and preserve the complex anatomy below the sylvian fissure. Conversely, stent-assisted coiling has been an important advancement in the endovascular treatment of MCA aneurysms and can allow the endovascular surgeon to overcome an unfavorable neck to dome ratio that Fields et al. and Regli et al. described as a common cause of embolization failure [45, 46]. However, use of a stent mandates dual antiplatelet therapy, and this has important ramifications when considering the need for potential CSF diversion. The Kuopio Cerebral Aneurysm Database found [3–6] that 29% of ruptured MBIF aneurysms presented with preoperative hydrocephalus. Abla et al. [32] found that 24.2% of patients presented with hydrocephalus. Obstructive hydrocephalus in the setting of aSAH obviates the need for an external ventricular drain (EVD) which increases the risk of dual antiplatelet therapy. Therefore, placement of an EVD and microneurosurgical clipping may circumvent the risk of hemorrhage associated with the EVD placement in the setting of dual antiplatelet therapy. On the other hand, placement of an external ventricular drain prior to initiation of dual antiplatelet therapy may be sufficient to avoid ICH which sustains the viability of endovascular repair in aSAH. Conversely, communicating hydrocephalus in the setting of aSAH lumbar drain is less problematic as CSF diversion can be accomplished in this setting with a lumbar drain which lowers the risk associated with hemorrhage.

## 5. Conclusion

Based on both the current literature and the collective experience of the authors, there is no absolute superiority with respect to coil embolization versus surgical clipping for the treatment of ruptured MBIF aneurysms. A strong case can be made for both treatment paradigms. The treating physicians must be cognizant of the multiple variables and complex nuances of vascular neurosurgery and consider each individual case in a multidisciplinary fashion. Microsurgical clipping remains an attractive option for MCA aneurysms given the ease of access via sylvian dissection, parent artery preservation, and durable occlusion. Expanding endovascular treatments such as endoluminal or intra-saccular technology may offer the surgeon a more robust armamentarium against these aneurysms. As things now stand, antiplatelet regimens limit their application in the setting of a rupture.
